# Outcomes of decompressive craniectomy for malignant middle cerebral artery stroke in an academic hospital in Brazil

**DOI:** 10.1055/s-0043-1772602

**Published:** 2023-10-04

**Authors:** Luiz Fernando Rodrigues de Oliveira, Millene Rodrigues Camilo, Luisa Franciscatto, Guilherme Gozzoli Podolsky-Gondim, Frederico Fernandes Alessio Alves, Rui Kleber do Vale Martins Filho, Francisco Antunes Dias, Koji Tanaka, Benedicto Oscar Colli, Octávio Marques Pontes-Neto

**Affiliations:** 1 Universidade de São Paulo, Faculdade de Medicina de Ribeirão Preto, Hospital das Clínicas, Departamento de Neurociências e Ciências Comportamentais, Ribeirão Preto SP, Brazil.; 2 Universidade de São Paulo, Faculdade de Medicina de Ribeirão Preto, Hospital das Clínicas, Departamento Cirurgia, Divisão de Neurocirurgia, Ribeirão Preto SP, Brazil.

**Keywords:** Stroke, Decompressive Craniectomy, Infarction, Middle Cerebral Artery, Acidente Vascular Cerebral, Craniectomia Descompressiva, Infarto da Artéria Cerebral Média

## Abstract

**Background** Ischemic stroke is an important cause of death in the world. The malignant middle cerebral artery infarction (MMCAI) has mortality as high as 80% when clinically treated. In this setting, decompressive craniectomy is a life-saving measure, in spite of high morbidity among survivors.

**Objective** To evaluate the outcomes of patients with MMCAI treated with decompressive craniectomy in a Brazilian academic tertiary stroke center.

**Methods** A prospective stroke database was retrospectively evaluated, and all patients treated with decompressive craniectomy for MMCAI between January 2014 and December 2017 were included. The demographics and clinical characteristics were evaluated. The functional outcome, measured by the modified Rankin Scale (mRS), was assessed at hospital discharge, after 3-months and 1-year of follow-up.

**Results** We included 53 patients on the final analysis. The mean age was 54.6 ± 11.6 years and 64.2% were males. The median time from symptoms to admission was 4.8 (3–9.7) hours and the mean time from symptoms to surgery was 36 ± 17 hours. The left hemisphere was the affected in 39.6%. The median NIHSS at admission was 20 (16–24). The in-hospital mortality was 30.2%. After a median of 337 [157–393] days, 47.1% of patients had achieved favorable outcome (mRS ≤ 4) and 39.6% had died.

**Conclusion** Decompressive craniectomy is a life-saving measure in the setting of MMCAI, and its effects remains important in the scenario of a middle-income country in real-world situations.

## Resumo

**Antecedentes** O acidente vascular cerebral (AVC) isquêmico é uma causa importante da morte em todo o mundo. O infarto maligno da artéria cerebral média (IMACM) tem mortalidade de até 80% quando tratado clinicamente. Nesse contexto, a craniectomia descompressiva é uma medida salvadora de vidas, apesar da alta morbidade entre os sobreviventes.

**Objetivo** Avaliar os desfechos dos pacientes com IMACM tratados com craniectomia descompressiva em um centro acadêmico terciário de AVC no Brasil.

**Métodos** Um banco de dados prospectivo de AVC foi avaliado retrospectivamente e todos os pacientes tratados com craniectomia descompressiva para IMACM entre janeiro de 2014 e dezembro de 2017 foram incluídos. As características clínicas e demográficas foram avaliadas. Os desfechos funcionais, medidos pela escala modificada da Rankin (mRS), foram avaliados na alta hospitalar, após 3 meses e após 1 ano de seguimento.

**Resultados** Foram incluídos 53 pacientes na análise final. A idade média foi 54,6 ± 11,6 anos e 64,2% eram homens. A mediana do tempo dos sintomas à admissão foi 4,8 (3–9,7) horas e o tempo médio dos sintomas à cirurgia foi 36 ± 17 horas. O hemisfério esquerdo foi o afetado em 39,6%. A pontuação na escala de AVC do National Institute of Health (NIHSS) à admissão foi 20 (16–24). A mortalidade hospitalar foi 30,2%. Após uma mediana de 337 (157–393) dias, 47,1% dos pacientes tinham atingido um desfecho favorável (mRS ≤ 4) e 39,6% tinham morrido.

**Conclusão** Craniectomia descompressiva é uma medida salvadora de vidas no contexto do IMACM e seus efeitos permanecem importantes no cenário de um país em desenvolvimento em situação de vida real.

## INTRODUCTION

Ischemic stroke remains the second leading cause of death and disability globally. 1 In middle income regions, such as Brazil and other Latin American countries, the lifetime risk of stroke is even higher and the number of deaths from stroke has increased in the last three decades. 2 3 Moreover, stroke care is not well stablished in most of these countries and the proportion of patients who receive any kind of recanalization therapy is still around 1%. 3 In this context, the management of patients with malignant middle cerebral artery infarction (MMCAI)—a very disabling entity that corresponds to approximately 10% of supratentorial strokes and has mortality as high as 80% when clinically treated—is of paramount importance. 4 5

Decompressive craniectomy (DC) was evaluated as a life-saving measure to alleviate brain swelling and improve outcomes in the setting of MMCAI in several European clinical trials and has been proved to be effective in preventing death and severe morbidity. 6 7 8 9 10 However, there are no randomized trials in developing countries and only a few studies evaluated outcomes of patients treated in these regions, most before the randomized trials era.

In this study, we aimed to evaluate the functional outcome of patients with MMCAI treated with DC at an academic tertiary stroke center in Brazil.

## METHODS

### Population

Patients were retrospectively selected from a stroke registry. The study was approved by the Ethics Committee of Hospital das Clínicas – Ribeirão Preto Medical School and all patients included on the stroke registry gave informed consent. We included all patients older than 18 years admitted between January 2014 and December 2017, who were treated with DC for management of MMCAI, defined as infarction on more than half of the middle cerebral artery territory. The exclusion criteria were: i) lacking enough information on the registry or medical records and ii) indication for the DC different from malignant edema (e.g. symptomatic hemorrhagic transformation).

### Clinical assessment

Patients with suspected stroke were assessed and treated according to Brazilian and international guidelines and the institutional protocol. 11 12 13 14 When eligible, intravenous thrombolysis and mechanical thrombectomy (MT) were performed. Those at risk of MMCAI were monitored preferably on intensive care unit or stroke unit and clinically reevaluated frequently. Additional imaging was performed according to the clinical discretion.

Eligibility to DC per local protocol included being younger than 60-years-old, clinical signs of total anterior circulation syndrome according to Bamford et al.'s classification, 15 reduced level of consciousness as scored on National Institutes of Health Stroke Scale (NIHSS) subitem 1a, and noncontrast computerized tomography (NCCT) evidence of involvement of more than 50% of MCA territory, or infarct size bigger than 145 cm ^3^ on the diffusion weighted imaging (DWI) sequence. The indication of surgical treatment followed the local protocol, along with the neurologist and neurosurgeon's joint decision. The surgical technique was decided according to the current practice. 16 17 18 Given the severity of the condition, some patients were treated beyond the predefined time and age limits, based on clinical judgment on premorbid condition, and neurological and imaging exams at the moment of the decision.

Upon hospital admission, clinical data were assessed and recorded, including NIHSS score and Glasgow coma scale (GCS), baseline systolic and diastolic blood pressure, and baseline serum glucose. The following demographic data were also systematically obtained: age, sex, cardiovascular risk factors, and previous functional status.

Time to events (brain imaging, thrombolysis, thrombectomy, and surgery) were recorded, as well as the type of recanalization therapy and site of arterial occlusion. The stroke etiology was classified according to the Trial of Org 10172 in Acute Stroke Treatment criteria. 19

The initial NCCT was performed at admission in all cases and retrospectively reviewed regarding arterial territory involved (only MCA or additional anterior or posterior cerebral artery territory involvement), the Alberta stroke programme early Computed Tomography score (ASPECTS), 20 hemorrhagic transformation and signs of brain herniation. When available, digital subtraction angiography and MT were also evaluated. The last imaging before surgery indication was also assessed according to the same criteria.

### Outcomes assessment

The modified Rankin scale (mRS) was assessed at discharge, 3-months and 1-year after stroke. In the cases which lacked data at 1-year, it was considered the closest date to 1-year available. Favorable functional outcome was defined as mRS ≤ 4.

Given the high morbidity of the disease and high dependency of survivors, it was also evaluated the patients' destiny after discharge: whether to home, rehabilitation hospital, or institutionalization at a support hospital.

### Statistical analysis

Continuous variables were described as central tendency measures and dispersion, means (± ) and standard deviations (SD), or medians and interquartile ranges (IQR). Categorical variables were shown as percentages. We used the Statistical Package Social Sciences version 24 (SPSS, IBM Corp. Armonk, NY) version 24 for all analysis. A *p* -value < 0.05 (two-sided) was used as the threshold for statistical significance.

## RESULTS

During the study period, 58 patients with supratentorial ischemic stroke treated with DC were identified. There were 4 patients excluded due to hemorrhagic transformation as the main indication for the surgery and other one due to the lack of information on the medical records. We included 53 patients on the final analysis. The mean age was 54.6 ± 11.6 years and 64.2% were men. At admission, the median NIHSS was 20 (16–24) and the median GCS was 12 (10–14). The right hemisphere was the affected in most cases (60.4%). The most common comorbidities were arterial hypertension (66%) and diabetes mellitus (32%).

The median time from symptoms to admission was 4.8 (3–9.7) hours and the median time from symptoms to surgery was 33.8 (24.7–44.2) hours. Among the patients, 45 (84.9%) were treated within 48 hours from symptoms onset. The complete clinical and demographics characteristics are summarized in Table 1 .

**Table 1:** Baseline clinical characteristics.

Characteristics ^a^		n = 53
**Clinical**	Male sex	34 (64.2)
	Age (years)	54.6 ± 11.6
	Time of admission to surgery (hours)	26.6 (14.6–37.0)
	Time of symptoms to surgery (hours)	33.8 (24.7–44.2)
	Length of hospital stay (days) ^b^	25 (14–46.5)
	Glucose at admission (mg/dL) ^b^	124 (109–179)
	Systolic blood pressure at admission (mmHg)	148 ± 27.7
	Diastolic blood pressure at admission (mmHg)	88 ± 17
**Neurological exam and brain imaging**	NIHSS at admission ^b^	20 (16–24)
	GCS at admission ^b^	12 (10–14)
	Left hemisphere	21 (39.6)
	ASPECTS at admission ^b^	6 (4–7.5)
	Midline shift before surgery (mm) ^b^	5 (2–7)
**Previous pathologic history**	Hypertension	35 (66)
	Diabetes mellitus	17 (32)
	Renal failure	2 (3.8)
	Atrial fibrillation	10 (18.9)
	Congestive heart failure	13 (24.5)
	Tobacco use	30 (56.6)
	Alcohol abuse	15 (28.3)
	Previous stroke	8 (15.1)
	Coronary disease	3 (5.7)
**Site of arterial occlusion**	Middle cerebral artery	20 (37.7)
	Intracranial internal carotid artery	11 (20.8)
	Tandem occlusion	14 (26.4)
	Unknown	8 (15.1)
**Recanalization therapy**	Intravenous thrombolysis	14 (26.4)
	Mechanical thrombectomy	18 (34)
**Stroke etiology**	Atherosclerosis	14 (26.4)
	Cardioembolism	16 (30.2)
	Other known causes	7 (13.2)

**Abbreviations:** ASPECTS, Alberta stroke program early computed tomography score; GCS, Glasgow coma scale; NIHSS, national institute of health stroke scale. **Notes:** ^a^ Values expressed as mean ± standard deviation or absolute number (percentage of total). ^b^ Values expressed as median (interquartile range).

We identified an in-hospital mortality rate of 30.2%. The surviving patients stayed in-hospital for 31 (18.5–42.5) days. At discharge, 16 (43.2%) patients went to home, 10 (27%) were discharged to rehabilitation hospital and then to home, and 11 (29.7%) were transferred to a support hospital, where they could receive nursing care and eventually could be discharged to a nursing home or their own.

During the in-hospital period, the most common complications were infectious, accounting for 75.5% of all patients. Acute kidney injury (37.7%) and seizures (24.5%) also had high prevalence. The in-hospital complications are listed in Table 2 .

**Table 2:** In-hospital complications.

Complications ^a^	n = 53
Seizures	24.5% (13)
Surgery-related complications	30.2% (16)
Urinary tract infections	18.9% (10)
Surgical site infections	24.5% (13)
Acute kidney injury	37.7% (20)
Pneumonia	58.5% (31)
Any infection	75.5% (40)
Venous thromboembolism	9.4% (5)

**Note** : ^a^ Values expressed as percentage (absolute number).

**Figure 1:**
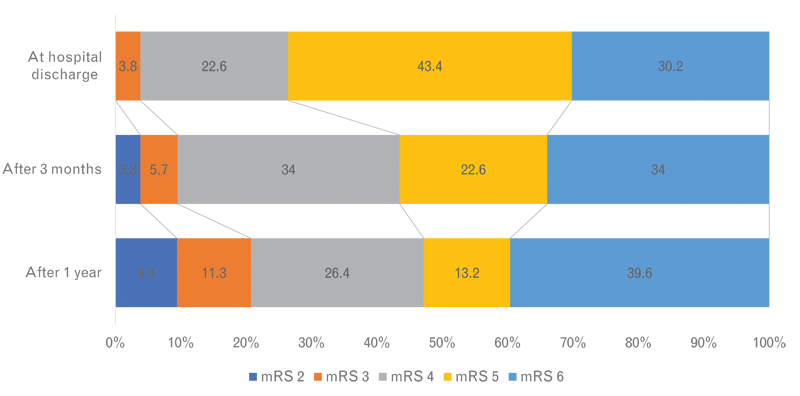
Modified Rankin scale (mRS) distribution at discharge, after 3-months, and after 1-year of stroke.

**Figure 2:**
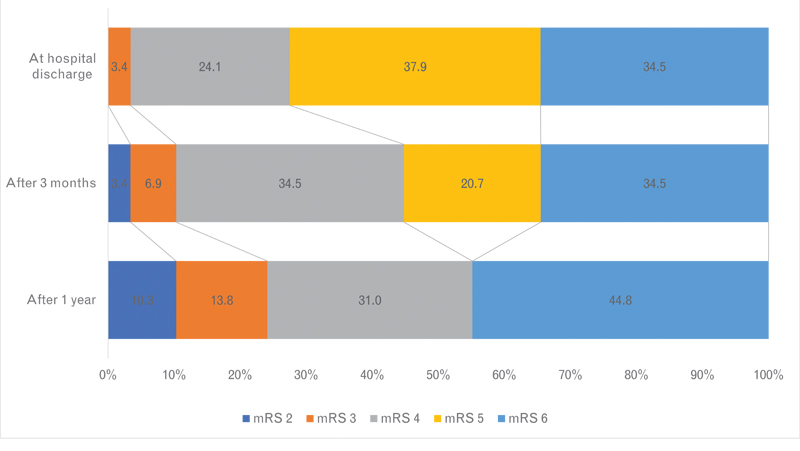
Modified Rankin scale (mRS) distribution at discharge, after 3-months, and after 1-year of stroke in the subgroup of patients younger than 60 years treated with decompressive craniectomy within 48 hours from symptom onset.

The functional outcome was described in Figure 1 . After a median of 337 (157–393) days, 25 (47.1%) patients had a favorable outcome (mRS ≤ 4). When considering only those patients aged 60 years or younger treated within 48 hours from symptoms onset (n = 29), the proportion of favorable outcome increases to 55.1% ( Figure 2 ). In this scenario, there was no survival with severe disability (mRS 5) and the mortality rate was 44.8%.

## DISCUSSION

Although the role of decompressive craniectomy on management of MMCAI has been studied in several randomized controlled trials (RCT) that showed its benefits on mortality and even on the prevention of severe disability, ethical and social concerns remain about when this procedure is recommended. 6 7 8 9 10 21 As shown on the metanalysis from the first three RCTs, up to 42% of survivals in the group of patients treated with DC had moderate to severe disabilities. 9 It is important to consider that these studies included only patients from high-income European countries and that there are unquestionably differences on socioeconomic aspects, as well as on the health care between European and middle-income countries, including Latin American ones. Those differences pose limitations for the extrapolation of data regarding treatment outcomes, especially regarding severe disabling diseases that requires continuous multidisciplinary attention and even financial support, such as stroke. There is only one RCT from a middle-income country that included patients older than 18 years without upper age limit, and it found that 58% of survivals in the surgery group had moderate to severe disabilities (mRS 4 or 5) after 12-months of follow-up. 21 However, the different inclusion criteria make it difficult to compare them.

In our study, we aimed to evaluate the outcomes of DC in real-world situations, considering the socioeconomics limitations inherent to a middle-income country. Our data shows similar distribution to those found on the first RCT. There was a reduction of the expected mortality rates if they had not been treated with DC at the expense of a high proportion of survivals with great disability. However, the magnitude of this effect appears to be more important on the RCT, as evidenced by the greater mortality (39 vs. 22%) and smaller proportion of survivals with mRS ≤ 3 (21 vs. 43%) in our population. 9

There are limited data about DC and stroke in middle-income countries. In the only study from Brazil that evaluated functional outcome measured by the mRS, Vital et al. included 60 patients, divided in two age groups (< 60 years and ≥ 60 years) and identified overall mortality of 55% after 90 days, while 38% were mRS ≤ 4 in the same period. 22 In China, Chen et al. described a population of 60 patients, with a 27% mortality rate at 1-year. 23 In a cohort of 31 patients with MMCAI treated with DC, 39% were dead after 1-year and 52% had mRS ≤ 4. 24 In India, Rai et al. evaluated 36 stroke patients treated with DC in a cohort of 60 patients and identified a mortality rate of 38% at 1-year and good outcome defined as mRS ≤ 4 in 53% of the patients, although the median time from symptoms to surgery was 56 hours. 25

Our results are similar to those found on these retrospective observational studies, but worse than the results found on RCT. In the pooled analysis of individual data from the first three RCT, 75% of the patients in the surgery group had mRS ≤ 4 and 22% were dead at 1-year of follow-up. 9 The worse outcomes can be attributed to several factors. The most obvious ones are the inclusion of patients beyond 48 hours from symptoms and older than 60 years, but some differences are a result of the real-world character from our study and from socioeconomic discrepancies between the countries involved, such as the inherent greater prevalence of comorbidities in our population and differences in health assistance and rehabilitation.

Limitations of our study include its retrospective design and the possible selection bias related to the moment of treatment decision, which might have led to the exclusion of patients with severe comorbidities and poor prognosis at baseline. However, our population and our results reflect a real-world scenario, with all its implications. Our follow-up time was longer than most retrospective studies in order to better reflect the continuous improvement of the surviving patients, and to offer a fair possibility of comparison to the randomized trials.

In conclusion, decompressive craniectomy is a life-saving measure in the setting of MMCAI, and its effects remains important in the scenario of a middle-income country in real-world situations. After 1-year of follow-up, there was a great reduction in expected mortality and an improvement in the proportion of surviving patients without severe disabilities.
